# ^90^Y/^177^Lu-DOTATOC: From Preclinical Studies to Application in Humans

**DOI:** 10.3390/pharmaceutics13091463

**Published:** 2021-09-13

**Authors:** Licia Uccelli, Alessandra Boschi, Corrado Cittanti, Petra Martini, Stefano Panareo, Eugenia Tonini, Alberto Nieri, Luca Urso, Matteo Caracciolo, Luca Lodi, Aldo Carnevale, Melchiore Giganti, Mirco Bartolomei

**Affiliations:** 1 Department of Translational Medicine, University of Ferrara, 44121 Ferrara, Italy; licia.uccelli@unife.it (L.U.); aldo.carnevale@unife.it (A.C.); melchiore.giganti@unife.it (M.G.); 2 Nuclear Medicine Unit, University Hospital, 44124 Ferrara, Italy; s.panareo@ospfe.it (S.P.); a.nieri@ospfe.it (A.N.); rsulcu@unife.it (L.U.); matteo.caracciolo@unife.it (M.C.); l.lodi@ospfe.it (L.L.); m.bartolomei@ospfe.it (M.B.); 3 Department of Chemical, Pharmaceutical and Agricultural Sciences, University of Ferrara, 44121 Ferrara, Italy; alessandra.boschi@unife.it; 4 Medical Physics Unit, University Hospital, 44124 Ferrara, Italy; e.tonini@ospfe.it; 5 Radiology Unit, University Hospital, 44124 Ferrara, Italy

**Keywords:** [^177^Lu]Lu-DOTATOC, [^90^Y]Y-DOTATOC, PRRT, neuroendocrine tumors

## Abstract

The PRRT (Peptide Receptor Radionuclide Therapy) is a promising modality treatment for patients with inoperable or metastatic neuroendocrine tumors (NETs). Progression-free survival (PFS) and overall survival (OS) of these patients are favorably comparable with standard therapies. The protagonist in this type of therapy is a somatostatin-modified peptide fragment ([Tyr3] octreotide), equipped with a specific chelating system (DOTA) capable of creating a stable bond with β-emitting radionuclides, such as yttrium-90 and lutetium-177. In this review, covering twenty five years of literature, we describe the characteristics and performances of the two most used therapeutic radiopharmaceuticals for the NETs radio-treatment: [^90^Y]Y-DOTATOC and [^177^Lu]Lu-DOTATOC taking this opportunity to retrace the most significant results that have determined their success, promoting them from preclinical studies to application in humans.

## 1. Introduction

The success of therapy in nuclear medicine is based on the increasing availability of radionuclides with adequate chemical-physical characteristics and of molecular probes capable of selectively transporting the radiation source into the tumor tissue. In the last ten years, over 80% of the publications relating to therapeutic radionuclides concern preclinical and clinical research conducted with conventional radionuclides, among which the most cited are ^131^I, ^90^Y, and ^177^Lu [1].

The key to their success lies in the recent and innovative nuclear medicine therapeutic strategy, which consists of the personalized theranostic approach.

Theranostics is the convergence point between diagnostic imaging and radiomolecular cancer therapy. There are several combinations of molecular targeting vectors and radionuclides suitable for theranostic use. Multi-element radiopharmaceuticals, consisting of two radioisotopes possessing similar chemical properties but having different physical emission properties, for example, ^99m^Tc/^188^Re [2,3,4], ^68^Ga/^177^Lu or ^68^Ga/^90^Y [5], used for the labeling of the same bioactive molecule, were the first and are still the most used in theranostic clinical practice. In this configuration, one radiopharmaceutical is used for therapeutic treatment of the tumor and the other one is used for diagnosis and response monitoring. [^68^Ga]Ga-DOTATOC combined with [^177^Lu]Lu-DOTATOC or [^90^Y]Y-DOTATOC are used in the theranostic model of Neuro Endocrine Tumors (NETs) radioreceptor.

The nuclear medicine research, in this particular therapeutic field, is constantly evolving thanks to the strong multidisciplinary synergy. In particular, the close collaboration of specialists from different disciplines such as physics, chemistry, radiochemistry, biochemistry, pharmacology, and nuclear medicine has determined the possibility to offer targeted therapies against solid neoplasms, such as NETs.

NETs include a heterogeneous group of neoplasms, exhibiting a variable biological behavior, that can originate from various organs with an estimated incidence of about 5 new cases per 100,000 individuals per year [6].

Overall, the highest incidence of these neoplasms is affecting the organs of the digestive system, in particular ileum and pancreas and, less frequently, stomach, duodenum, colon and appendix, constituting the gastro-entero-pancreatic NET (GEP) and representing 60–70% of all NETs. In addition to GEP NETs, other histotypes, affecting, in particular, the respiratory system and bronchi (20–30%) or other organs (10%) such as skin, thyroid, parathyroid, thymus, paraganglia and adrenal glands, can be classified as non-GEP NETs [7,8,9].

The overall 5-year survival of NET patients is on average about 67.2% and can vary from 15% to 95% depending on the site of origin, the extent of the disease, and cellular biological characteristics [10]. The correct diagnostic approach of NETs is based on a careful evaluation of the clinical history, on the identification of general and specific biohumoral markers and on the localization of the primary tumor and any metastases through endoscopic and echo-endoscopic, morphological (CT or MRI), and functional (SPECT-CT with [^111^In]In-pentetreotide or PET-CT with [^68^Ga]Ga-SST-As) [5].

To date, there are no specific and consolidated therapeutic protocols to be routinely employed in the treatment of NETs. Nevertheless, there are many therapeutic options for this pathology and most patients with NET have the possibility of obtaining a good response to treatments and a good prognosis. Consequently, the management and therapy of these patients should be approached by clinicians with a multidisciplinary approach: a multimodal therapeutic strategy should be designed from the time of diagnosis and for each individual patient, rather than simply delivering an empirical sequence of treatments. The therapeutic approach will depend on the location of the primary tumor, the histological examination, the stage, and the grade of the neoplasm. Diagnosis of NETs often occurs in an advanced stage of the disease, given the non-specificity of the symptoms and the general slow progression, making the surgical approach for curative purposes applicable in the minority of cases.

Since the liver is the organ with the highest incidence of metastases, alternative treatments to surgery such as locoregional approaches including transarterial chemoembolization (TACE) [11] and Selective Internal Radiation Therapy (SIRT) [12] should be considered. In case of metastatic spreading, numerous systemic therapies are available including therapy with somatostatin analogues in long-acting release (LAR) form [13], chemotherapy [14], up to the most innovative target therapies such as biological therapies (e.g., mammalian target of rapamycin (mTOR) inhibitor drugs) and the peptide receptor radionuclide with radiolabeled somatostatin analogues (PRRT) [15,16,17,18].

NETs plasmalemma shares the expression of specific molecules, proteins and receptors that make up the neuroendocrine phenotype. Some of these biomarkers have been extensively studied as specific targets for targeted diagnostic and therapeutic approaches.

In particular, NETs almost constantly over-express membrane receptors capable of binding, with high affinity, somatostatin (SST) [19,20]. To date, five somatostatin receptor subtypes have been identified (SST-R1, SST-R2, SST-R3, SST-R4, SST-R5), and the SST-R1, SST-R2, SST-R3 subtypes are the most expressed by NET cells [21,22].

However, the SST (cyclic neuropeptide consisting of 28 amino acids, secreted by neurons and cells of the endocrine system) cannot be used as a bio-probe for the aforementioned receptors due to its instability and its very short half-life.

The availability of synthetic analogues of SST (SST-As) and of synthesis strategies, allowing SST-As to be stably—in vitro—labelled to specific low and high energy radioisotopes, has allowed the development of specific radiopharmaceuticals and consequently innovative theranostic nuclear medical pathways. By these radiopeptides, it is currently possible to selectively convey radiation in tumor lesions expressing somatostatin receptors (SST-Rs), both for diagnostic and therapeutic purposes [23,24].

The most used radiopharmaceuticals in PRRT are those deriving from the combinations of the two isotopes yttrium-90 and lutetium-177 and of the two somatostatin analogues DOTATOC and DOTATATE: [^90^Y]Y-DOTATOC, [^90^Y]Y-DOTATATE, [^177^Lu]Lu-DOTATATE, [^177^Lu]Lu-DOTATOC. However, the greatest clinical experience (and the most abundant scientific literature) refers to the use of [^90^Y]Y-DOTATOC, which represented the first generation radiopharmaceutical for NETs. More recently, the [^177^Lu]Lu-DOTATATE was used in the phase 3 Netter-1 study [25], which prompted the production and commercialization of the Lutathera^®^. In recent years, a smaller number of trials involved the use of [^90^Y]Y-DOTATATE while the interest and use of [^177^Lu]Lu-DOTATOC is still growing [26,27].

In 2016, waiting for the availability of Lutathera^®^, our group designed an experimental protocol for therapeutic treatment that included all types of NETs, both GEP NETs and non-GEP NETs. Starting from the diagnostic phase performed with the SST analogue DOTATOC radiolabeled with ^68^Ga ([^68^Ga]Ga-DOTATOC), we proposed both [^90^Y]Y-DOTATOC and [^177^Lu]Lu-DOTATOC as therapeutic radiopharmaceuticals [28,29,30,31].

In order to obtain the necessary authorizations from the national competent authorities, the study protocol was accompanied by the specific Investigational Medicinal Product Dossier (IMPD) concerning the two radiopharmaceuticals included in the study protocol (FENET-2016, EUDRACT number: 2016-005129-35, NCT04790708). To address the various issues concerning the compilation of the IMPD, an extensive literature review of the last 25 years was necessary to allow us to retrace the main steps relating to the PRRT preclinical and clinical studies performed with DOTATOC labeled with high energy isotopes.

In particular, in this review, the characteristics of the most widely used therapeutic radiopharmaceuticals for the treatment of NETs and the path that led them successfully from preclinical studies to application in humans will be described.

The purpose of this review is, therefore, to perform an accurate analysis of the literature and make it available for the clinical and scientific community.

## 2. PRRT: Radionuclides and Radiopharmaceuticals

The ability to selectively transport radiation to tumor tissue for a therapeutic purpose by means of peptides is the goal of receptor-mediated radiometabolic therapy and the NETs represent an “ideal” neoplasms group for PRRT, since most of these tumors have a slow evolution and a high expression of somatostatin receptors (SST-Rs). PRRT is a therapeutic modality that has been tested in humans for about 25 years, and still today stimulates an active interest and a continuous planning of studies aimed at identifying the most suitable radiopharmaceuticals in terms of efficacy and safety and to find the appropriate PRRT placement in the complex therapeutic algorithm of NETs.

The pharmacokinetic, pharmacodynamic, and therapeutic aspects of radiolabeled SST-As, as well as their efficacy and safety profiles, can be better understood if the characteristics of the specific cancer target, the chemical, physical, and biological characteristics of the radiopharmaceuticals, as well as the mechanism of their interaction are known.

**Figure 1:**
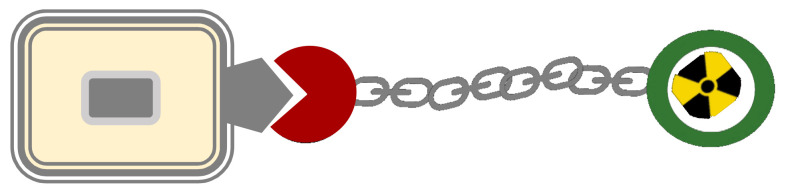
Schematic representation of peptide receptor radionuclide with radiolabeled somatostatin analogues (PRRT) therapeutic model. This is based on the existence of a target, overexpressing SST-Rs and a radiopharmaceutical, consisting of peptide which have a high affinity for the SST-Rs expressed by the target, an adequate radioactive isotope and a chelator able to stable bond the radionuclide.

This particular PRRT therapeutic model (Figure 1) is based on the existence of a target, consisting of neoplastic lesions that overexpress SST-Rs, in particular type 2 and 5 receptors (SST-R2 and SST-R5) and a radiopharmaceutical consisting of a peptide having high affinity for the SST-Rs expressed by the target, an adequate radioactive isotope and a chelator capable of guaranteeing an indissoluble bond between them determining the accuracy of therapy. In these radiopharmaceuticals the pharmacokinetics and pharmacodynamics and consequently the therapeutic power is strongly influenced, when the same radionuclide is used, by the type of peptide, especially as regards the biodistribution, the uptake in the target lesions and in the remaining non-target and critical organs.

The affinity for the SST-Rs is peculiar to each specific SST-As considered [19,22]. Most peptides currently in clinical use have an agonist action and are able, after interacting with the receptor, to be stably internalized within the cell, and it is here that the isotope linked to exerts the therapeutic effect through a mechanism of action based on the release of radiation. The radiation released is responsible for the effectiveness of the treatment, but also for its toxicity.

Table 1 shows SSt-As, radioisotopes and chelating systems most currently used in clinical PRRT. All SSt-As ligands are based on octapeptides such as octreotide (OC), [Tyr_3_ ]octreotide (TOC), [Tyr_3_, Thr_8_ ]octreotide (TATE) and [1-Nal_3_] octreotide (NOC), with peculiar SST receptors affinity. Focusing the attention on the chemical characteristics of these ligands, it was found that the replacement of Phe_3_ in octreotide by Tyr_3_ leads to an improved SST-R2 affinity, but the SST-R3 and SST-R5 affinity is reduced; the C-terminal introduction of Thr for Thr(ol) in TATE results in a SST-2- selective ligand with a 7-fold improvement of SST affinity. The NOC derivative (1-Nal in position 3) leads to a peptide with affinity to SST2,3,5. [32].

DOTATOC consists of a peptide ([DOTA0-Tyr3]-octreotide or SMT487 or Edotreotide), somatostatin analogue, functionalized with an amide bonded to a chelating bifunctional macrocycle DOTA (acid 1,4,7,10-azaciclododecan-*N*,*N*′,*N*″,*N*‴-tetraacetic), able to coordinate the radionuclides ^90^Y or ^177^Lu, under controlled reaction conditions.

**Figure 2:**
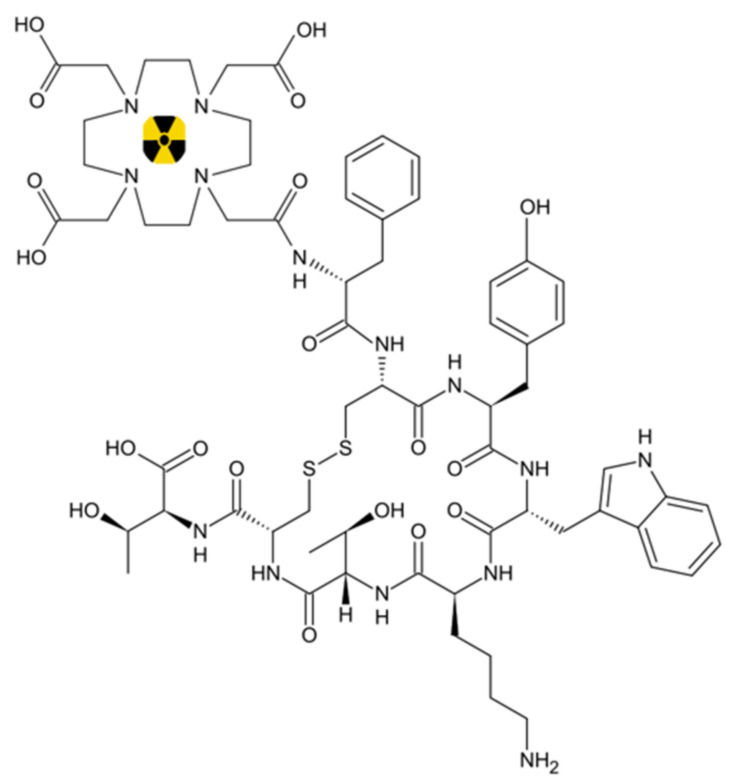
Chemical structure of [^90^Y]Y-DOTATOC and [^177^Lu]Lu-DOTATOC = ^90^Y or ^177^Lu.

The peptide moiety (TOC), representing the “VECTOR” component, is responsible for the pharmacokinetics and pharmacodynamics of the final radiopharmaceuticals [^90^Y]Y-(1,4,7,10-tetraazacyclododecane-N,N,N,N-tetraacetic acid)-Tyr3-octreotide ([^90^Y]Y-DOTATOC, Figure 2) and [^177^Lu]Lu-(1,4,7,10-tetraazacyclododecane-N,N,N,N-tetraacetic acid)-Tyr3-octreotide ([^177^Lu]Lu-DOTATOC, Figure 2).

DOTATOC has higher affinity for type 2 somatostatin receptors (SST-R2), with IC_50_ = 14 ± 2.6 nM, and lower for type 5 (SST-R5) and type 3 (SST-R3), with IC_50_ = 393 ± 84 nM and IC_50_ = 880 ± 324 nM respectively [19,22].

The ^90^Y and ^177^Lu radioisotopes represent the “EFFECTOR” component of the [^90^Y]Y-DOTATOC and [^177^Lu]Lu-DOTATOC radiopharmaceuticals respectively.

**Figure 3:**
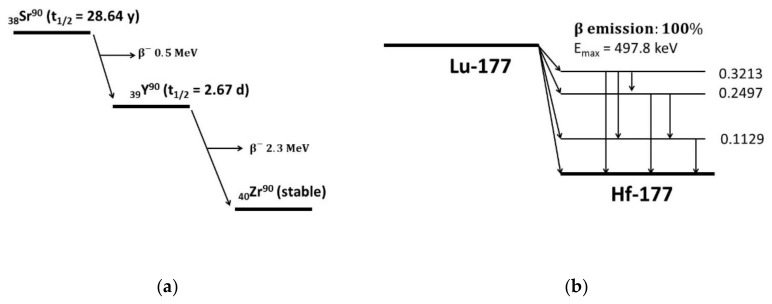
Decay schemes of ^90^Y (**a**) and ^177^Lu (**b**).

Because of their specific physical profile, ^90^Y and ^177^Lu are responsible for the selective irradiation of the target and collateral irradiation of critical organs (Table 2 and Figure 3).

^90^Y and ^177^Lu are both radiometals having different physical characteristics.

The pharmacokinetic and pharmacodynamic behavior of these radiopharmaceuticals is mainly determined by the functionalized peptide, regardless of the radioisotope with which it is coordinated (^111^In, ^68^Ga, ^86^Y, ^90^Y or ^177^Lu). It follows that the pharmacological characteristics of [^111^In] In-DOTATOC, [^68^Ga-]Ga-DOTATOC, [^86^Y]Y-DOTATOC, [^90^Y] Y-DOTATOC and [^177^Lu]Lu-DOTATOC can be considered almost superimposable, especially for the last two radiopharmaceuticals [23,33,34].

The first and fundamental studies relating to the pharmacological and dosimetric characteristics of [^xx^X]X-DOTATOC were performed in humans after radiolabeling with ^86^Y (t_1/2_ = 14.74 h) and ^111^In (t_1/2_ = 2.83 d) [35,36]. The ^86^Y isotope is a positron emitter that guarantees good PET imaging and accurate quantitative assessments; its short physical half-life, however, does not allow to obtain the essential delayed data for a complete dosimetric evaluation. The isotope ^111^In, on the other hand, possesses chemical-physical characteristics (long physical half-life, adequate gamma emission) ideal for studying the pharmacological profile (absorption, distribution, elimination, etc.) and for obtaining the necessary dosimetric evaluations and dose estimates for target lesions and critical organs.

The data obtained from the administration of [^86^Y]Y- or [^111^In]In-DOTATOC were used to predict the in vivo behavior of radiopharmaceuticals based on the use of DOTATOC radiolabelled with β^-^ isotopes with higher energy emitters such as ^90^Y and ^177^Lu [37,38]. Due to the different emission characteristics associated with the two radionuclides, it is to be expected the absorbed dose to tumor was higher for ^90^Y as for ^177^Lu after administration of the same level of radioactivity and the ^90^Y radiopharmaceuticals were more effective on large lesions, while those with ^177^Lu on smaller lesions.

[^90^Y]Y-DOTATOC was the first of the two radiopharmaceuticals to be produced as described in the review by Otte and collegues (1998) showing that, when administered systemically, [^90^Y]YDOTATOC stably binds to neoplastic lesions expressing SST-Rs [39]. Therefore, following a cellular internalization process, the radiopharmaceutical is able to deliver high quantities of radiant energy (see physical characteristics of ^90^Y) to the target, causing apoptosis and death of pathological cells.

Some organs (hepatic and splenic parenchyma) physiologically accumulate the radiopharmaceutical, regardless disease presence. The amount of free radiopharmaceutical is eliminated through the intestine and, in particular, the kidney. In the renal system, precisely at the level of the proximal convoluted tubule, the radiopharmaceutical is reabsorbed by the same tubular cells by an active transport system [40,41].

This last process can cause radiotoxicity to the organ. The pre and post intravenous administration of amino acids (L-lysine and/or L-arginine), through a competition mechanism, inhibits the radiopharmaceutical amount reabsorbed at the tubular level and, therefore, the dose absorbed by the kidney to a variable extent from 9 to 53% [42,43].

[^177^Lu] Lu-DOTATATE was the first radiopharmaceutical of ^177^Lu used in PRRT and subjected, starting from the 2000s, to phase II clinical studies. Consequently, the greatest pharmacological knowledge refers to this radiopharmaceutical, so much so that today it is the only radiopharmaceutical approved by the competent authorities for [^177^Lu] Lu-DOTATATE. DOTATATE, as mentioned above, has a different affinity (compared to DOTATOC) for SST-R2 [32], and this affects its pharmacological characteristics and especially as regards its distribution at the level of target lesions and other organs, including the critical organs.

### Preparation of [^90^Y]Y-DOTATOC/[^177^Lu]Lu-DOTATOC

**Figure 4:**
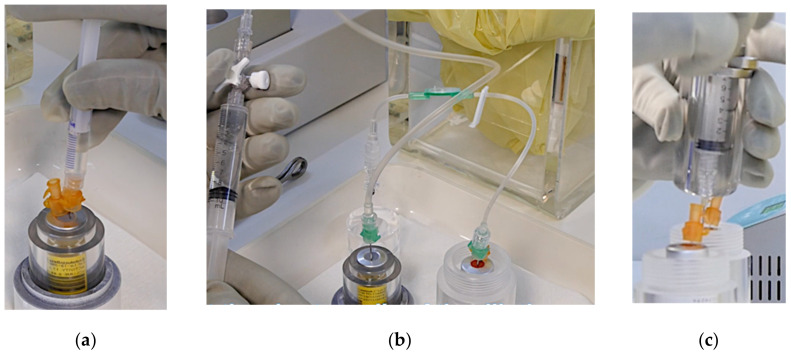
[^177^Lu]Lu-DOTA-TOC and [^90^Y]Y-DOTATOC synthesis (**a**), dilution and sterilization (**b**) and manual fractionation (**c**).

Despite the many advantages of automating radiopharmaceutical manufacturing processes, e.g., improved radiation protection, repeatability, traceability etc., only a few complete studies on the automation of the peptide or antibody labeling process with 𝛽^−^- radionuclide emitters are available, and they are limited to specific ligands or radionuclides [44]. The preparation of [^90^Y]Y-DOTATOC or [^177^Lu]Lu-DOTATOC is still mainly performed using manual procedures although the radioactivity managed during the synthesis and fractionation of these radiopharmaceuticals is very high. Normally, the radiopharmaceutical product is a 0.9% NaCl/buffer solution (e.g., ascorbate buffer) containing an activity between 16.65 and 24.05 GBq in a total volume approximately 18 to 26 mL. The radioactive concentration of the solution is on average about 0.92 GBq/mL. However, regardless of the production method adopted, the synthesis and quality control procedure is the same and is described hereafter in accordance with European Pharmacopoeia. According to the revised procedure [45], [^177^Lu]Lu-DOTA-TOC and [^90^Y]Y-DOTATOC were obtained conjugating the [^177^Lu]LuCl_3_ or [^90^Y]YCl_3_ with the DOTATOC peptide in the acetate form. The radiopharmaceutical synthesis must be carried out in a classified isolator. Both radiopharmaceuticals can be prepared by adding into the reactor, containing the radioisotope [^177^Lu]LuCl_3_ (or [^90^Y]YCl_3_), the solubilized peptide in ascorbate buffer (Figure 4a). At the end of the incubation (about 30 min at 90 °C) a small amount of the solution is used to perform quality control (pre-release: appearance, identification, chemical purity, radiochemical purity, radionuclide purity, pH; post-release: sterility and pyrogenicity).

The solution containing the radiopharmaceutical can be suitably diluted with physiological solution (normally an aliquot of 1 mM DTPA is added at the end of the labeling having the function of chelating agent for free ^90^Y or ^177^Lu), sterilized (0.2 mm sterile filter) (Figure 4b) and finally fractionated (Figure 4c). The administration of the radiopharmaceutical can be executed via peripheral intravenous injection. In order to obtain adequate hydration and to protect kidneys during the radiopharmaceutical excretion, all patients should be administered with arginine. Both during the administration of the radiopharmaceutical and for the following 48 h, the patients should be hospitalized in special isolation rooms.

The action mechanism of the two radiopharmaceuticals, as already mentioned, begins with the interaction between the radiolabeled peptide and the somatostatin receptor placed on the cell membrane followed by the cellular internalization of the complex. The therapeutic efficacy depends on the physical characteristics of the radionuclide, in particular, the presence of ^177^Lu makes the radiopharmaceutical more suitable for the treatment of small lesions (≤20 mm) while the ^90^Y makes it more suitable for the treatment of large lesions (>20 mm) [29].

Since many researchers have produced and collected pharmacokinetic and pharmacodynamic data, comparing the different radiopharmaceuticals belonging to this radiopeptides category, these data will be presented in the following paragraphs with particular reference to [^177^Lu]Lu-DOTATOC and [^90^Y]Y-DOTATOC.

## 3. Preclinical Studies of Radiolabeled DOTATOC

The main preclinical pharmacology and toxicology studies on radiolabeled somatostatin analogues for diagnostic and therapeutic purposes were mainly conducted by Dutch and Swiss researchers in the late 1990s and early 2000s. In most of these works, the various aspects related to the pharmacodynamics, pharmacokinetics, and toxicity of the various somatostatin analogue radiolabeled peptides (especially DOTATOC with indium-111 and yttrium-90 and less with gallium-68 and lutetium-177) in animal models were addressed and discussed.

In a study conducted in 1994 by Taylor et al. [46] the presence of SST-R2 was demonstrated in animal models. High concentrations of these receptors have been found in pancreatic and lung cancer cell lines and lower concentrations in breast, prostate, melanoma and liver cancer cells. They employed the cyclic hexapeptide somatostatin analogue [^125^I]MK-678 to evaluate the expression of surface membrane receptors and found that the binding of somatostatin peptides with high SSTR2 affinity and antiproliferative properties were powerful inhibitors of [^125^I]MK-678 binding to various tumor species, indicating that they can practice antitumor effects via the SSTR2 receptor.

De Jong et al. [47], in one of their first studies in animal models, showed that DOTA, compared to DTPA, a chelator commonly used for octreotide labelling with indium-111 ([^111^In]In-DTPA] octreotide, OctreoScan^®^; London, UK), was the ideal chelator for the radiosynthesis of radiolabeled somatostatin analogues. Moreover, a new modified octreotide Tyr3-octreotide (TOC), conjugated with the DOTA chelator (DOTATOC) and radiolabeled with indium-111 or yttrium-90 showed greater affinity for tumor cells expressing receptors for somatostatin with respect to [^111^In-DTPA0]-octreotide] and, therefore, a high diagnostic and therapeutic potential, respectively.

The potential nephrotoxicity following therapeutic use of radiolabeled somatostatin analogues was already known after the first pharmacological and scintigraphic studies with radiopeptides. In an animal study conducted by Bernard et al. [40], it was shown how the intravenous administration of L- and D-lysine in aqueous solution is able to significantly reduce the undesirable uptake of the radiopeptide in the renal parenchyma of male Wistar rats. In particular, this study demonstrated that the kidney uptake of [^90^Y]Y-DOTATOC has decreased by 65% intravenous administrated D-lysine, while the radioactivity of the blood, the pancreas and the adrenal glands were not affected. The D-lysine may be preferred to the L-lysine for reducing renal absorption of radioactivity during scintigraphy and PRRT due to lower toxicity and its disturbing with the metabolic equilibrium of natural amino acids.

In a study published the following year, de Jong et al. [48] confirmed that the co-infusion of D-Lysine is able to reduce by more than 50% the undesired uptake of radiopeptide in the renal parenchyma and, consequently, the dose absorbed to the kidney and toxicity, without alterations in the distribution of radioactivity in the blood and target organs.

In the same year, Stolz et al. [49] published the first work, which clearly demonstrates the therapeutic efficacy of the analogue [DOTA-dPhe1,Tyr3]octreotide labeled with yttrium-90 in animal models carrying tumors expressing somatostatin receptors. The study conducted on Lewis rats carrying pancreatic tumor CA 20948 expressing SST-R2 affirmed that: (1) [^90^Y]Y-DOTATOC binds rapidly to the tumor of rats; (2) 24 h after administration, the ratio of radiopharmaceutical concentration in the tumor to blood is equal to 49.14; (3) a single administration of 10 mCi/kg of [^90^Y]Y-DOTATOC is able to determine a complete response in 5/7 experimental animals, with no signs of recurrence disease in the following eight months.

In a fundamental study by Froidevaux et al. [50] in 1999, some somatostatin analogues such as DOTA^0^-(D) Phe^1^-octreotide (DOTAOc), DOTA^0^-(D) Phe^1^ Tyr3-octreotide (DOTATOC), DOTA^0^-(D)^®^Nal1-lanreotide (DOTALan) and DOTA^0^-(D) Phe^1^-vapreotide (DOTAVap) were tested, compared and better defined from a pharmacological point of view radiolabeled with indium-111, iodine-125 and yttrium-90 and tested in mouse models carriers of AR4-2J tumor with high SST-R2 receptor expression. For all DOTA-peptides included in the study, regardless of the incorporated isotope type, favorable biodistribution profiles were observed in animals with: (1) high receptor affinity for SST-R2 membrane receptors located on AR4-2J tumor cells; (2) a rapid clearance of radiopeptides from all tissues not expressing SST-R2 with the exception of the renal parenchyma and a specific uptake of radiopeptides in tissues expressing SST-R2 and in particular in tumor lesions; (3) excellent intra-tumor penetration of DOTA-peptides, and of DOTATOC in particular, which correlates with a high diagnostic and therapeutic efficacy.

To get these results, chelator-free peptides [Oc and TyrOc (or TOC)], DOTA-chelated peptides (DOTATOC) and peptides conjugated with non-radioactive yttrium were tested, taking into account that the incorporation of the metal, radioactive or not, could influence the receptor affinity of the complex. The results obtained showed that the affinity (expressed as IC_50_) of TOC, DOTATOC and Y-DOTATOC (0.33, 2.44, 2.37 nM) for SSR-T2 has the highest values compared to the other analogs and that the bond with DOTA influences the binding affinity between the TOC peptide and the SST-R2, while the further incorporation of the metal inside the DOTA does not involve changes in receptor affinity.

To better define the therapeutic potential and the potential toxicity of the radio-peptides under examination, the tumor/liver, tumor/muscle, tumor/kidneys and tumor/blood ratios were evaluated 4 h after administration. The values relating to DOTATOC were found to be the highest, and therefore more advantageous from a radiodosimetric point of view compared to the other radiopeptides.

The distribution kinetics of the various radiopeptides were tested in animals by means of scintigraphic images collected with indium-111 over a period of time ranging from 4 to 48 h after administration. The maximum tumor uptake was detected 4 h after administration for all radioligands, including DOTATOC, for the latter together with DOTALAN, the highest uptake compared to OctreoScan was detected. Furthermore, DOTATOC showed the lowest tumor clearance value (62% of the maximum tumor uptake was still present at 24 h) and, therefore, the highest tumor retention value in the 48-h observation period. Renal distribution was maximal at 4 h after administration and for DOTATOC was relatively low compared to the other peptides.

A valid indicator of efficacy and safety for the therapeutic use of radiopeptides has been identified in the ratio between the surface area (under the distribution curve in the period 4–48 h after administration) relative to the tumor and the surface of the tumor area (under the distribution curve in the period 4–48 h after administration) relative to the renal parenchyma. The tumor/kidney ratio of DOTATOC was, once again, more advantageous than the other radioligands (1.15 for DOTATOC, 0.33 for DOTAOc, 0.29 for DOTALan, 0.22 for DOTAVap and 0.16 OctreoScan).

This study has therefore shown that, among the DOTA-peptides examined in animals, DOTATOC has the best pharmacological profile, and it is probably the most suitable and promising radioligand for nuclear medical oncological clinical applications.

Later in 2001, de Jong et al. [51] evaluated the therapeutic effect of [^90^Y]Y-DOTATOC in CA20948 pancreatic tumors, of various sizes, transplanted into Lewis rats. The study showed the ability of ([^90^Y]Y-DOTA(0),Tyr(3))octreotide to control tumor growth, especially in medium-sized tumors and included in a volumetric range ≥1 cm^2^ and ≤14 cm^2^. The effect of radionuclide therapy appeared to be dependent on tumor size at the onset of therapy. They confirmed the therapeutic effect of [^90^Y]Y-DOTATOC previously demonstrated by studies Stoltz et al. [49].

The therapeutic potential of somatostatin analogues radiolabeled with the β-emitters ^90^Y (high energy) and ^177^Lu (low energy) have been evaluated in rat model by de Jong [51] and coworker in 2001. The authors employed [^90^Y]Y-DOTATOC and [^177^Lu]Lu-DOTATATE in rats with tumor lesions (CA20948 or AR42J) expressing somatostatin receptors ranging in size from 0.1 to 15 cm^2^. The animals were treated with 1–2 intravenous administrations (one week apart) of 277.5–555 MBq of [^177^Lu]Lu-DOTATATE or 379 MBq of [^90^Y]Y-DOTATOC and followed for at least 150 days after therapy. They observed that the antitumor response obtained depended on the size of the lesions and, in particular, that ^177^Lu was more suitable for small lesions while ^90^Y showed greater efficacy on large lesions. These studies subsequently paved the way for clinical trials reaching conclusions still valid and applied in the most modern clinical protocols of PRRT [52].

Subsequently, Capello et al. [53] evaluated the therapeutic effects of D-Phe-c(Cys- Tyr-D-Trp-Lys-Thr-Cys)-Thr(ol) (Tyr 3-octreotide) and D-Phe-c(Cys-Tyr-D-Trp-Lys-Thr-Cys)-Thr (tyr3-octreotate) somatostatin analogs chelated with tetra-azacyclododecatatro-acetic acid (DOTA) and labeled with ^90^Y or ^177^Lu in an in vitro colony-forming assay using the rat pancreatic tumor cell line CA20948. This study showed that these radiopeptides, to an extent dependent on the activity of the radiopharmaceutical used, were able to cause, even definitively, the arrest of tumor growth.

### 3.1. Pharmacodynamics Studies

The somatostatin analogue radiolabeled peptides pharmacodynamics is substantially determined by the carrier component represented by the peptide, which, on the basis of its intrinsic and specific affinity for somatostatin receptors, binds to the target. The isotope, incorporated in the ring of the chelating molecule (which in turn is stably conjugated to the peptide), represents the effector component and is responsible for the therapeutic action that takes place through the deposition of ionizing radiation at the target level. The therapeutic effect occurs after intracellular internalization of the receptor-radiopeptide complex and varies in relation to the type of isotope used.

Primary pharmacodynamics is determined by the interaction between the radio-labeled DOTATOC and the SST-R2 receptor, with consequent formation of the radio-peptide-receptor complex and its internalization at the level of the neoplastic cells over-expressing SST-R2 [46]. The action of primary pharmacodynamics is responsible for the therapeutic effect of the radiopeptide [47].

Secondary pharmacodynamics is determined by (i) the interaction between radiolabeled DOTATOC and SST-R2 receptor, with consequent formation of the radio-peptide-receptor complex and its internalization at the level of the cells that physiologically express SST-R2, present in the spleen, in the liver and in the pituitary; (ii) the distribution of a portion of radiolabeled DOTATOC in the bloodstream and, in part, in the hematopoietic marrow; (iii) from the re-uptake of a portion of radiolabeled DOTATOC at the level of the proximal convoluted tubule of the nephron. The aforementioned actions related to the secondary pharmacodynamics of the radiopeptide are related to unwanted irradiation to healthy/critical organs: the extent of this irradiation varies depending on the type of isotope used [43,47,50].

The experiments conducted on animals have shown that the pharmacodynamics of radiolabeled DOTATOC is favorable based on the rationale of the therapeutic models studied. Pharmacodynamic data collected on animals have also been confirmed in human studies.

### 3.2. Pharmacokinetics Studies

The radiolabeled DOTATOC pharmacokinetics was studied in animal models, mainly murine, after systemic administration of the radiopeptide, to evaluate its plasma kinetics, its distribution in the tumor target and in healthy/critical organs and the modalities of excretion.

Tumor cell lines expressing SST-R2 were injected or transplanted to reproduce the lesions in animals. The plasma kinetics of the radiopeptides used were studied by means of serial blood samples over time and by means of urine collection. To evaluate the distribution of the radiopharmaceutical at the level of tumor lesions and healthy and potentially critical organs, the various tissues and organs were isolated from the sacrificed animals. Quantitative assessments of body fluids, tissues and organs were performed by direct radioactivity count (MBq/g), scintigraphic images and autoradiographic techniques [53].

The study conducted by Froidevaux and coworkers [50] showed that the systemic intravenous administration of the radiolabeled DOTATOC ensures complete and optimal absorption of the radiopeptide within the blood compartment: this determines the achievement of a rapid equilibrium with the extracellular fluid and rapid diffusion into tissues and organs. Blood clearance is equally rapid. The radiolabeled DOTATOC has a high rate of tumor uptake with maximum uptake 4 h after administration. Furthermore, DOTATOC showed the lowest tumor clearance value (62% of the maximum tumor uptake was still present at 24 h) and, therefore, the highest total morality retention value in the 48 h observation period in comparison with DOTA0-(D) βNal1-lanreotide (DOTALan), and DOTA0-(D)Phe1-vapreotide (DOTAVap). Renal distribution was maximal at 4 h after administration and relatively low compared to other peptides.

To the best of our knowledge, no studies in animals to evaluate the metabolism and any degradation products of the radiolabeled DOTATOC are available. This state has been researched and studied in humans by Cremonesi et al. [36] and by Otte et al. [39]. Both authors report that, by analysis with FPLC, the absence of radiopeptide degradation products can be demonstrated in blood samples up to 3 h after administration, and in urine up to 8 h after administration. In late urine samples, the presence of traces of ^111^In-DOTA-DPhe was detected. The elimination of the radiolabeled DOTATOC not fixed to the target occurs almost exclusively through the renal emunctorium. It has also been shown in animals that the kidney is the critical dose-limiting organ in this therapeutic model. The possibility of reducing renal toxicity by systemic administration of amino acids has been documented in numerous animal dosimetry studies [43,54].

The experiments conducted on animals have shown that the pharmacokinetics of radiolabeled DOTATOC is favorable based on the rationale of the therapeutic models studied. The pharmacokinetic data collected on animals have also been confirmed in human studies.

### 3.3. Toxicological Studies

The potential toxicity of radiolabeled DOTATOC is not attributable to the peptide (for low quantities used) but to the type and activity of the isotope used. ^90^Y, due to its physical characteristics, is able to deliver energy to the target and to healthy organs about 3 times greater than ^177^Lu [55]. Considering the length of the radiation path (about 10 mm for ^90^Y and about 4 mm for ^177^Lu) it can be understood how the toxicity data are variable based on the different volumes that the single analogous organs reach in the various animal species and in man [56].

The toxicity profile of DOTATOC was assessed—as such and after radiolabeling with ^90^Y—in acute toxicity studies, intravenously, in mice and rats, and in late toxicity studies, intravenously, lasting four weeks in rats and non-human primates. Mutagenic potential was evaluated in vitro (Ames test, chromosomal aberration in human lymphocytes, and mutation assay in mouse lymphoma cells) and in vivo (micronucleus). Local tolerability of the solution for injection was investigated in an intravenous local irritation study in rabbits. LD50 values of administered intravenously DOTATOC were >150 mg/kg (450 mg/m^2^) in mice and 59 mg/kg (354 mg/m^2^) in rats. Single intravenous administrations of [^90^Y]Y-DOTATOC at doses of 120, 240, 360 mCi/m^2^ have documented a no-observed-effect-level (NOEL) value in monkeys at 120 mCi/m^2^. The NOEL values in rats treated daily for 4 weeks were: 1 mg/kg/day (6 mg/m^2^/day) and 0.3 mg/kg/day (1.8 mg/m^2^/day) in males and females, respectively. The NOEL values in monkeys, treated intravenously daily, for 4 weeks, were: 0.3 mg/kg/day (3.6 mg/m^2^/day) and 1 mg/kg/day (12 mg/m^2^/day) in males and females, respectively [53].

## 4. Clinical Studies of Radiolabeled DOTATOC

### 4.1. Pharmacokinetics and Pharmacodynamics Studies

The DOTATOC pharmacokinetics and biodistribution have been studied in humans after labeling with ^86^Y [35] and with ^111^In [36]. The data obtained from these studies were of fundamental importance since their transposition on therapeutic models with ^90^Y and ^177^Lu allowed one to obtain accurate predictions in terms of absorbed dose to tumor lesions and healthy/critical organs and efficacy and safety profile. The evaluation of the kinetics of the system is carried out using a multi-compartmental developed by Cremonesi et al. [36] to interpret the biokinetics from experimental data on biodistribution of [^111^In]In-DOTATOC [36]. In the multi-compartment model, which involves the intravenous systemic administration of the radiopeptide, an optimal and complete absorption of the radiolabeled DOTATOC within the blood compartment is assumed and the achievement of rapid equilibrium with the extracellular fluid compartment (ECF). The authors demonstrated that the clearance of [^111^In]In-DOTATOC from the blood compartment is very rapid and the circulating rate decreases to less than 9 ± 5% within the first hour and to less than 0.9 ± 0.4% within 10–12 h of intravenous administration.

Regardless of a possible district intra-arterial administration, which could determine a change in the distribution to the target based on the “first-pass” effect and which finds application in the localizations of disease with predominantly hepatic localization [57,58] no formulations to be used through the oral route.

Jamar et al. [35] with [^89^Y]Y-DOTATOC, obtained superimposable pharmacokinetic data and demonstrated that: (i) there is a rapid disappearance of the radiopeptide from the bloodstream with a plasma residue of the injected activity of approximately 5% after 5 h and ≤1% at 24 h; (ii) the circulating activity is entirely confined to plasma with a share linked to red blood cells of less than 1%; (iii) the fraction of stable radiopeptide circulating in the plasma is high (95.3 ± 4.7%) and that this value remains constant over time (with observations up to 3 h after administration) confirming the effectiveness of the chelating molecule DOTA; (iv) intravenous co-infusion of amino acids (L-Lysine and or L-Arginine) for the protection of the renal parenchyma did not significantly modify the plasma clearance curve of [^89^Y]Y-DOTATOC.

Kwekkeboom et al. [59] documented that the plasma distribution pattern of [^177^Lu]Lu-DOTATATE is comparable to that of [^111^In]In-DTPA-octreotide, with rapid scintigraphic visualization of the renal parenchyma after administration and of the liver, spleen, kidneys, (and in some patients pituitary and thyroid) and tumor lesions 4 h after administration. The authors also reported in their study that the distribution patterns of the two radiopeptides at 24 h after administration are similar, with corresponding uptake on the liver, kidney and spleen; on the other hand, tumor lesions are more intensely (up to 3–4 times) and longer uplifting after administration of [^177^Lu]Lu-DOTATATE. From the percentage biostribution data obtained, the absorbed dose values for critical organs and tumor lesions were extrapolated. For the latter, it was demonstrated, by theoretical model, that the absorbed dose was 4 times higher for [^177^Lu]Lu-DOTATATE compared to [^111^In]In-DTPA-octreotide and 2 times higher for [^177^Lu]Lu-DOTATATE compared to [^90^Y]Y-DOTATOC [39].

Esser et al. [38] compared [^177^Lu]Lu-DOTATATE with [^177^Lu]Lu-DOTATOC. For both, the clearance in the blood is very rapid and the plasma concentration is less than 10% of the activity administered after 3 h. During the first 24 h the clearance of [^177^Lu]Lu-DOTATOC is slightly faster than that of [^177^Lu]Lu-DOTATATE but this difference is not statically significant and, therefore, the clearance is evaluated as overlapping for the two radiopeptides.

As regards the residence time τ in tumor lesions and in healthy/critical organs, however, a significant difference has been documented which, with the same clearance times and in the presence of the same isotope and the same chelating molecule, is imputable—as expected—to the different receptor affinity that the two distinct somatostatin analogues (TOC and TATE) have for SST-R2. In particular, the tumor τ value is significantly longer for patients treated with [^177^Lu]Lu-DOTATATE, with a mean ratio of 2.1:1 compared to [^177^Lu]Lu-DOTATOC. Similarly, the value of τ is in the ratio of 1.4:1 and 1.5:1 for the renal and splenic parenchyma, respectively. The authors conclude that, based on residence times, a higher dose absorbed to the tumor but also a higher absorbed dose to the renal parenchyma is foreseeable in the case of [^177^Lu]Lu-DOTATOC, in the case of [^177^Lu]Lu-DOTATOC a lower absorbed dose to the tumor, but also a lower dose absorbed to the kidney which, however, remains the critical and limiting organ in PRRT.

Conversely, in a more recent study, Schuchardt et al. [27] demonstrated that [^177^Lu]Lu-DOTATOC delivers the lowest doses to healthy organs and has a more advantageous ratio—in terms of absorbed dose—between tumor and kidney, compared to [^177^Lu]Lu-DOTATATE and [^177^Lu]Lu-DOTANOC (Table 3). Other studies report similar data and believe that [^177^Lu]Lu-DOTATOC is a radiopeptide with excellent safety and efficacy profiles in PRRT and, certainly, not lower than [^177^Lu]Lu-DOTATATE [38].

The elimination of the unbound portion of radiolabeled peptide occurs mainly through the renal emunctorium and, to a lesser extent, through the intestinal emuntorium. The amount of radiopetide present in the pre-urine undergoes reabsorption (mediated by an active transport mechanism) at the level of the proximal convoluted tubule cells. This reabsorption—if a somatostatin analogue radiolabelled with ^90^Y or ^177^Lu is used—causes undesirable irradiation to the renal parenchyma (3.3 ± 2.2 mGy/MBq, in the case of [^90^Y]Y-DOTATOC, and equal to 0.8 mGy/MBq for [^177^Lu]Lu-DOTATATE and 0.6 mGy/MBq for [^177^Lu]Lu-DOTATOC) which deserves extensive consideration in the dosimetric evaluation, in order to limit renal damage. The deposit of radioactive urine at the level of the bladder cavity is a source of irradiation of the wall of this organ (2.21 ± 0.31 mGy/MBq, in the case of [^90^Y]Y-DOTATOC): this situation must also be taken into consideration, especially for when it concerns the aids to stimulate and facilitate diuresis.

From the studies carried out, it has been shown that the amount of radiopetide eliminated through the intestinal emuntorium is reduced to the limits of significance and therefore determines a negligible dosimetric load. With [^111^In]In-DOTATOC it was estimated that the cumulative activity of radiopeptide eliminated in urine was 52% ± 12% of the amount administered 4 h after administration and 73 ± 11% after 24 h from administration. Although the radioexposure of the renal parenchyma in the case of [^177^Lu]Lu-DOTA-SST-As is lower than the values reported in PRRT with [^90^Y]Y-DOTA-SST-As, it is still essential to consider the kidney as a critical organ and apply all available aids (evaluation of provisional dosimetry and administration of amino acids) in order to limit the damage.

The prevalent renal excretion of the radiopeptide was confirmed with [^86^Y]Y-DOTATOC [60,61]. It has also been seen that the cumulative excretion values can present wide variations (29–96% of the administered activity) and that these depend on the number of lesions present and their uptake index. More than 50% of the initial activity was found in the urine eliminated within the first 3.5 h of administration; the percentage of excretion between the 24th and 48th hour was equal to 4.4%. It has also been confirmed that the cumulative excretion of the radiopeptide is significantly higher in the case of co-infusion of amino acids for nefro-protective purposes. As further confirmation of the efficiency of the DOTA chelator, it was found that up to 24 h after administration, 98.6 ± 2.0% of the radioactivity present in the urine consisted of peptide still stably radiolabeled.

Esser et al. [38] showed that the cumulative activity eliminated in the urine is higher for [^177^Lu]Lu-DOTATOC compared to [^177^Lu]Lu-DOTATATE with percentages equal to 81% and 71%, respectively.

### 4.2. Safety and Effectiveness Studies

In two decades of follow-up, relating to numerous clinical studies, a lot of information was extracted about the safety and efficacy of PRRT. In terms of safety, PRRT is a well-tolerated treatment, with absent or mild-moderate toxicity in most cases, if performed with the correct prescribing methods and adopting all measures aimed at limiting the absorbed dose to the renal parenchyma and to the hematopoietic marrow, which represent the critical dose-limiting organs. In terms of efficacy, PRRT is able to determine an objective response with a reduction in size of lesions and tumor-specific markers, a reduction in symptoms and therefore an improvement in the quality of life and, overall, an increase in survival times.

The use of ^177^Lu in PRRT was introduced in 2000, with the somatostatin analogue DOTATATE peptide. The use of [^177^Lu]Lu-DOTATATE has spread rapidly and—especially due to its potential better profile of renal toxicity due to the physical characteristics of the isotope used—has relegated the use of ^90^Y radiolabeled peptides to the background. The first studies with [^177^Lu]Lu-DOTATATE were carried out at the Erasmus University of Rotterdam. The key work regarding PRRT with [^177^Lu]Lu-DOTATATE is that of Kweekeboom et al. [62], which includes 504 patients, 310 of whom were considered to evaluate the efficacy of the treatment that involved intravenous administration of 7.4 GBq of [^177^Lu]Lu-DOTATATE, for 4 consecutive administrations, with intervals of 8 weeks. As regards the objective response—measured with the SWOG criteria—the following results were obtained: CR: 2%, PR: 28%, MR: 16%, SD: 35%. In the same population, the median OS was found to be greater than 48 months and the PFS equal to 33 months. Direct comparison with a series of published data from a similar population estimated that patients undergoing PRRT with [^177^Lu]Lu-DOTATATE achieved an estimated survival benefit of 40–72 months. Although these data are not derived from a randomized study, the difference in survival reflects the real therapeutic impact that PRRT can have in the care of NET patients. The authors state that the data relating to the response obtainable from PRRT can compare well with those relating to other treatments, such as chemotherapy. In this study, it was also observed that the best therapeutic responses were obtained in those patients who showed high lesional uptake at baseline scintigraph with OctreoScan^®^ and limited liver disease. In contrast, disease progression was more frequent in patients with low performance status and extensive liver disease. An attempt to categorize the objective response on the basis of histology allowed us to understand that NETs of pancreatic origin had a tendency to respond better than other GEP NETs and that functioning forms (pancreatic gastronomy) had a tendency to relapse earlier than to the remaining histotypes included in the study. In this study, the authors evaluated toxicity in 504 patients. Acute side effects that appeared within 24 h of radiopharmaceutical administration included nausea, vomiting and abdominal pain (10–25% of administrations). Grade 3–4 hematological toxicity (WHO), although transient, affected 9.5% of patients. Episodes of severe late toxicity (renal failure, hepatic failure and myelodysplastic syndrome) involved 13 patients (2%).

On the experience of the Dutch group, other European Centers have carried out PRRT studies with [^177^Lu]Lu-DOTATATE: these studies (Table 4) although related to less numerous samples, report substantially similar results to those obtained in the study previously reported. In all the works, the problem relating to renal toxicity is re-proposed which, although it is reduced compared to the experiences with analogues ^90^Y radiolabeled, is not, however, negligible in the case of use of analogues radiolabeled with ^177^Lu. The results of the NETTER1 study [25] is the first randomized phase 3 study in the field of PRRT. As expected, and demonstrated in previous studies of the last 20 years, the NETTER1 study reaffirms the potential of PRRT in interfering with neoplastic activity and, more importantly, in increasing patient survival. Table 4 summarizes the main studies in which the radiopeptide [^177^Lu]Lu-DOTATATE was used for the treatment of NETs of the gastro-entero-pancreatic tract (GEP-NETs) of bronchial NETs (B -NETs) and other histotypes (some not strictly NETs but still expressing SST-R2). In particular, the following are summarized: the sample of treated patients, the histological variant, the objective response, the survival times, the toxicity profile and the most relevant clinical conclusions.

[^177^Lu]Lu-DOTATOC is another radiopeptide used in PRRT [77]. It has been shown that, compared to DOTATATE, despite having a lower affinity for somatostatin receptors—and for SST-R2 in particular—DOTATOC has a shorter residence time inside the kidney and, consequently, the absorbed dose to the renal parenchyma could be lower using the latter peptide [38].

Among the various studies in the literature, the phase 2 study by Baum et al. [26] is relevant and exhaustive. The authors evaluated the efficacy and safety profile of [^177^Lu]Lu-DOTATOC used in patients with advanced NETs. 56 patients with metastatic and progressive disease were studied (50%: GE-NET, 26.8%: P-NET, 8.9%: B-NET, 14.4: other less frequent forms) and treated with various cycles (1-4) of [^177^Lu]Lu-DOTATOC, with a median activity per cycle of 7.0 GBq and with intervals of 3 months between one cycle and the next. The results of this study confirmed that [^177^Lu]Lu-DOTATOC determines an objective response and survival times comparable to those obtained using [^177^Lu]Lu-DOTATATE. The most interesting thing that emerges from the results is the absence of significant side effects, especially affecting the renal parenchyma. 20% of the patients included in the study and treated presented (even before the PRRT) moderate renal insufficiency: in these patients, an objective lesion response was obtained, and no worsening related to the pre-existing renal deficit was manifested. Table 5 summarizes the main studies in which the radiopeptide [^177^Lu]Lu-DOTATOC was used, as the only radiopharmaceutical or in association with [^90^Y]Y-DOTATOC, for the treatment of NETs and other histotypes (some not strictly NETs but still expressing SST-R2). In particular, the following are summarized: the sample of treated patients, the histological variant, the objective response, the survival times, the toxicity profile and the most relevant clinical conclusions.

The analysis of the reported studies concerning the use of [^177^Lu]Lu-DOTATOC (also in association with [^90^Y]Y-DOTATOC), shows a high percentage of objective responses and high survival times. Furthermore, the safety profile is also favorable, with bone marrow toxicity percentages comparable (and in some cases lower) to those reported in the PRRT studies with [^177^Lu]Lu-DOTATATE.

Trials that compared the various radiopharmaceuticals used in PRRT, (both ^90^Y and ^177^Lu based) showed that ^90^Y radiopharmaceuticals are more effective on large lesions, while those with ^177^Lu on smaller lesions [28]. From these evaluations arose the tendency to devise protocols in which patients affected by NET could be treated with combined regimens of radiopharmaceuticals, both based on ^90^Y and ^177^Lu [30,31].

These experimental studies have also largely rehabilitated the use of Yttrium-90 in PRRT [87,88].

### 4.3. Toxicological Studies

Acute and late side effects can be distinguished. Acute adverse events occur within 24 h after administration of the radiopharmaceutical: they are generally mild in intensity and resolve within a few days. Among these, nausea and, more rarely, vomiting are related to the concomitant administration of amino acids and are usually grade 1–2 [89].

The administration of the radiopharmaceutical can cause or exacerbate any carcinoid syndrome, more or less typical, caused by the sudden massive release of peptides or by receptor hyperstimulation. This syndrome is characterized by the type of peptide involved (for example: hypo/hypertension, hypo/hyperglycemia, sweating, tachycardia, skin flushing, hypergastrinemia, more or less intense diarrhea up to WDHA) can lead to prolongation of hospitalization but it is controllable with adequate patient support [90]. Another symptom that often occurs is the sensation of generalized asthenia, more or less accentuated, which accompanies the patient for 20–30 days following the administration of the radiopharmaceutical [91] and a slight accentuation of the physiological hair loss (observed after administration of ^177^Lu) [92]. Sub-acute and chronic adverse effects on critical organs—affecting the hematopoietic marrow and renal parenchyma—have a potentially greater impact on patient management, but as already mentioned, they are generally moderate and manageable if appropriate treatment protocols are adopted, which involve a preliminary dosimetric evaluation and, in particular, the co-administration of molecules able to reduce irradiation to the renal parenchyma [93]. A not uncommon sub-acute manifestation is represented by hematological toxicity, which occurs early, within 4–6 weeks after PRRT, is caused by an irradiation of the bone marrow and is generally mild to moderate and transient. Major toxicity pictures (WHO grade 3 or 4) can arise in <15% of patients who, more or less rapidly, show recovery of hematopoietic function [94]. Severe and permanent pictures of bone marrow toxicity are rare events since, in this type of treatment, the dose absorbed in the hemopoietic marrow is certainly below the toxicity threshold. Nevertheless, there have been some sporadic cases of myelodysplastic syndrome and acute myeloid leukemia [95].

However, renal toxicity remains the predominant aspect regarding the safety of PRRT. Often the suffering of the renal parenchyma is late and permanent and is related to the exceeding of certain thresholds of administered activity [96]. Several retrospective dosimetric studies reviewed the large number of patients treated with PRRT (both ^90^Y and ^177^Lu) and concluded that they were mainly patients with a history of diabetes and arterial hypertension (or who had already undergone nephrotoxic treatments such as chemotherapy) those that most frequently presented, after PRRT, signs and symptoms correlated with renal toxicity. The population was therefore divided into a subpopulation of subjects with risk factors. In which it was not recommended to deliver a dose to the kidney greater than 28 Gy, and into a second subpopulation of patients, who did not present risk factors and who could receive higher doses but, nevertheless, within the predetermined limit of 40 Gy at the renal parenchyma. Subsequent dosimetric calculations, based on the above threshold values, made it possible to extrapolate the maximum levels of cumulative activity and per cycle to be administered in the two distinct categories of patients [97]. In PRRT, the amount of radiation delivered to lesions and healthy organs depends both on the type and amount of isotope used and on the type of peptide associated with it. It has been estimated that, for the same administered activity, the absorbed dose to the tumor and critical organs varies in a 3 to 1 ratio, depending on whether peptides radiolabeled with ^90^Y or ^177^Lu respectively are used [45]. The degree of affinity between receptor and type of peptide directly affects the residence times of the respective radiopeptide within the target lesions and critical organs. Most authors agree on the need to perform a preventive dosimetric evaluation in all patient candidates for PRRT [98].

The purpose of the dosimetric study is to provide patient-specific information by evaluating the maximum activity that can be administered to the patient with the aim of maximizing the dose to the tumor and minimizing the dose to healthy organs by evaluating, in the specific case of PRRT, the risk of hematological and renal toxicity by estimating the dose absorbed by these organs. Other dosimetric and clinical studies underline the importance of fractionation (over time) of the cumulative activity of the radiopharmaceutical, as an operative modality capable of ensuring greater therapeutic efficacy and lower risk of toxicity [99].

With the greater experience acquired by the various PRRT Centers, cases of nephrotoxicity are currently a rare event, even if, despite the protocols of nephroprotection, a detriment of renal function can be manifested, which can be quantified in a reduction in clearance creatinine of about 7.3% per year after [^90^Y]Y-DOTATOC and 3.8% per year after [^177^Lu]Lu-DOTATATE [100]. Furthermore, it has been observed that the decline in renal function is more rapid for patients with the previously mentioned risk factors.

The PRRT, beyond its effectiveness in terms of objective response and survival, is able, according to some studies, to improve the quality of life of patients: this is especially true for those patients affected by secreting tumors with syndrome typical of neuroendocrine tumors (insomnia, inappetence, diarrhea, skin flushing) and/or for those in which the disease is very extensive (abdominal and/or skeletal pain) [101,102].

## 5. PRRT: Risks, Benefits, and Considerations

The nuclear physician who is preparing to treat a patient with PRRT must, in advance, carry out a careful analysis of the benefits and risks related to the treatment. To do this, it is necessary first of all: (i) to proceed to a correct selection of the patient to be treated and, therefore, (ii) carefully consider the type of radiopharmaceutical and the therapeutic scheme to be proposed based on the characteristics of the patient and a preliminary dosimetric evaluation, so as to guarantee personalized treatment, (iii) operate within a multidisciplinary group and in line with the main national and international guidelines [62,103]. The appropriateness level of the initial choices will be decisive for obtaining adequate results in terms of efficacy, safety, and sustainability.

The PRRT benefits are based on its documented therapeutic efficacy which, in practical terms, manifests itself through the interference with tumor growth and the reduction/resolution of lesions, the increase in survival times and the improvement of the quality of life, in patients with NET and also with other histotypes expressing somatostatin receptors. PRRT has been used with benefit in clinical settings in which diffuse and metastatic lesions were present, in those characterized by residual lesions after surgical treatment (adjuvant purpose) and in situations in which an inoperable solitary primary lesion was present (neoadjuvant purpose). In certain situations, the PRRT has also documented greater therapeutic benefits compared to chemotherapy treatments and medical therapies based on the use of biological drugs. In other studies, however, a benefit of PRRT in combination with other drugs has been documented.

The risks of PRRT are related to its potential side effects, which, as already mentioned above, can be acute, subacute, and often reversible or chronic and, therefore, permanent.

The Council Directive 2013/59/Euratom fixes the need for personalized dosimetry to patients treated with radionuclide therapy. In order to fulfil such a directive, an absolute quantification of the activity in the targets of the treatment and the organs at risk for each subject is necessary. The personalization of radiometabolic therapy passes from the knowledge of the dose to the organs and therefore from their uptake.

The starting point for such aim is a precise estimate of the spatial resolution and the sensitivity of the gamma camera exploited for the SPECT-CT studies, as well as an improvement in the uncertainty assessment associated to the measurements and the image reconstruction. The critical organs to monitor and preserve during PRRT are the hematopoietic marrow and, above all, the renal parenchyma, which is unduly irradiated by the amount of radiopeptide that does not bind to the target and which, through a re-uptake mechanism, enters the cells tubular section of the proximal nephron. Patients with labile hepatic compensation will also need to be carefully evaluated before being treated with PRRT.

The factors that can lead to predicting a benefit obtainable with PRRT and the factors that, on the other hand, can be correlated with a potential risk of toxicity are summarized and commented on below.

PRRT benefit predictors are [18,62,71]:

(a) factors related to the biological characteristics of the tumor:
  (a1) Elevated lesional expression of SST-R2.
  In fact, a high receptor expression is essential to ensure adequate accumulation of radiopharmaceuticals and a consequent adequate radiation dose to tumor lesions. Positive imaging with [^111^In]In-pentetreotide and mostly [^68^Ga]Ga-SS-As guarantee high accuracy to obtain this type of information. Numerous recent studies have confirmed that the lesion level uptake index (assessable by applying the Rotterdam scale in the monophotonic survey with [^111^In]In-pentetreotide and in a semi-quantitative mode by measuring the SUV in the PET-CT survey) is potentially correlated with the magnitude of the objective response (OR) and the efficacy of the treatment in terms of overall survival (OS) and progression-free survival (PFS), reductions in symptoms and, therefore, improved quality of life (QoL).
  (a2) Histology positive for NET.
  NETs include the neoplasms that most frequently and most abundantly express SST-R2. Among these, the histological variants that statistically best respond to PRRT include those of the gastro-entero-pancreatic tract and the forms of broncho-pulmonary origin. NETs that originate in the remaining organs of the respiratory system and in other locations (skin, thyroid, CNS, meninges), as well as other histotypes with neuroendocrine phenotypes, generally have less responsiveness and efficacy to the treatment.
  (a3) Well-differentiated low graded shapes (WHO) [104].
  The histotypes with a high degree of differentiation, i.e., G1 (Ki67 ≤ 3%) and G2 (Ki67 ≤ 10%), provide the best profiles of objective response and efficacy in terms of survival at PRRT. Some histotypes with G2 (Ki67 > 10%) and especially the well-differentiated G3 (Ki67 > 20%) forms may respond to treatment but have lower PFS and OS values.
  (a4) Limited spread of disease.
  The extent of disease spread is inversely proportional to the degree of objective response and therapeutic efficacy in terms of PFS and OS. Very often the NETs are indolent and slowly progressive, and their diagnosis occurs frequently when the disease is already systemic due to the presence of diffuse metastases or in any case not surgically attacked. Where technically possible and the patient’s general condition permits, surgical or interventional procedures aimed at eradicating or reducing the disease are recommended. Adjuvant post-surgical therapies, including PRRT, may be more successful after tumor debulking.
  (a5) Hepatic and pancreatic localization.
  Secondary hepatic lesions from NET are those that most frequently respond to PRRT, but primary NETs of the pancreas also show good responses to treatment. More resistant to treatment are secondary lymph node lesions and, above all, skeletal ones. PRRT with intra-arterial administration of the radiopharmaceutical through the hepatic artery represents—in patients with localized liver disease—an alternative modality (compared to the classic systemic intravenous administration) capable of expanding the therapeutic response and outcome of patients.
  (a6) Good performance status.
  A good general clinical status of the patient, a good life expectancy and the absence of comorbidities are potential factors directly related to the success of the treatment. In particular, the absence of risk factors for bone marrow toxicity (anemia, leukocytopenia, thrombocytopenia from previous chemotherapy or radiation treatments) and for renal toxicity (diabetes, hypertension, primary and secondary nephropathies) allows, in a context of greater tolerability by of these two critical organs, the administration of the highest levels of administrable radiopharmaceutical activity, which results in a higher absorbed dose to the tumor lesions.
  (a7) Favorable genotype.
  It is known, in clinical practice, how similar clinical presentations of NETs can respond differently or even opposite to the various types of treatment, including PRRT. The evaluation of the cellular genome, which can be performed with specific tests still in the experimental phase (NeTest), will, in the near future, describe the state of the disease and predict its prognosis and possible response to therapeutic treatments [105,106].
(b) Factors related to the therapeutic management of the patient to be treated:
  (b1) Multidisciplinary evaluation.
  The preliminary discussion of each single clinical case, the collegial choice of treatment, the timing and sequencing between the different treatments that make up the patient’s therapeutic plan, as well as the participatory evaluation of the follow-up, represent the ideal prerequisites for an efficient and effective management of the patient affected by NETs. Various studies correlate patient outcomes with how they are managed within structured specialized clinical paths and/or specialized and highly equipped Centers to respond to this type of patient.
  (b2) Adherence to PRRT protocols (and guidelines).

The shared guidelines IAEA-EANM-SNMMI [18], which draw from the numerous clinical dosimetric works reported in the previous paragraphs, provide specific indications about the treatment schemes to be used in PRRT. These reference documents explain the radiopharmaceutical activities (per cycle and cumulative) to be used in the various categories of patients (with or without risk factors), the interval between the various cycles, and the most suitable type of radiopharmaceutical. The various protocols are outlined that involve the use of somatostatin analogues (DOTATOC/DOTATATE) radiolabeled with ^90^Y for large lesions, with ^177^Lu for small lesions or combined or sequential treatments. By now, the hydration schemes to be performed collateral to the therapeutic treatment are known, including those relating to the infusion of nephroprotective molecules. Furthermore, personalized dosimetry is always recommended—in compliance with the optimization principle—in order to make the treatment as effective and safe as possible. The MIRD scheme provides the recognized and validated technical lines for dosimetry in nuclear medical therapy and, in particular, the OLINDA/EXM software is widely used to estimate the absorbed dose to the target and critical organs after administration of ^90^Y and ^177^Lu-labeled peptides.

PRRT risk predictors are [18,62,71]:

(a) Reduced bone marrow function.

Adequate bone marrow reserve should be present in PRRT candidates. Reference values recommend: WBC > 3000/μL, with absolute neutrophil value >1000/μL, PLT > 75,000/μL for [^177^Lu]Lu-DOTATOC and >90,000/μL for [^90^Y]Y-DOTATOC, RBC > 3,000,000/μL. Previous myelotoxic chemotherapy treatments and extensive radiotherapy treatments on the bone marrow (to the pelvis and spine), especially if performed in the weeks preceding the PRRT, increase the risk of bone marrow toxicity after PRRT. In cases of suspected haematological compromise, it could be useful to perform a biopsy of the haematopoietic marrow to verify the pre-PRRT situation, in order to evaluate the possible risks that the PRRT itself could bring and therefore implement the precautions aimed at reducing potential risks (reduction of the activity to be administered, longer interval between one cycle and the next). In any case, the overall bone marrow dose should always be ≤2 Gy. In relation to the activities (^90^Y or ^177^Lu) administered, the persistence of low platelet values, after the first courses of treatment, could affect the ability to recycle within the scheduled time and administer the planned activities at the start of treatment. Severe acute toxicity, albeit reversible, was reported in less than 10–13% of patients treated with ^90^Y and in 2–3% of those treated with ^177^Lu. In addition, sporadic cases of acute myelodysplasia and leukemia have been described.

(b) Reduced kidney function.

The kidney represents the dose-limiting organ for radiopharmaceutical activities normally used in PRRT. Therapy with ^90^Y is recommended in situations in which renal function (normalized for the patient’s age) is preserved. Treatments with ^177^Lu can also be admitted to patients with mild renal function impairment but, in any case, with creatinemia values ≤1.7 mg/dL. Renal parenchyma protection based on the administration of amino acids (L-Lysine and/or L-Arginine) before, during and after PRRT treatment is always recommended in all patients, regardless of starting renal function. For this concomitant treatment, attention should be paid to the possible hyperkalemia [107,108].

Some chronic diseases such as uncompensated diabetes, uncontrolled hypertension, obstructive nephropathies, as well as any previous nephrotoxic chemotherapeutic treatments (based on platinum) represent risk factors for the development of a potential toxicity induced by PRRT. On the basis of these observations, the absorbed dose to the kidney, in terms of BED (biological effective dose), was set at a threshold value of ≤40 Gy for the standard population and ≤28 Gy for subjects with a positive history of aforementioned pathologies favoring the onset of renal toxicity. Although the clinical experience accumulated in the last twenty years by the main PRRT centers has considerably reduced the occurrence of side effects, the most recent summary data confirm the possibility of a deterioration of renal function, more accentuated in the PRRT protocols in where ^90^Y labeled peptides are used compared to those based on the use of analogues conjugated with ^177^Lu.

Absolute contraindications to PRRT [62]: pregnancy; severe concomitant acute illness; severe psychiatric disorders.

According to what emerges from the literature and clinical experience, it can be said that PRRT constitutes an essential tool for the treatment of numerous patients with neuroendocrine neoplasia. In particular, taking into consideration the data concerning the potential toxicity and the documented therapeutic efficacy, the balance between the risks and the benefits clearly leans in favor of PRRT.

## Figures and Tables

**Table 1:** Ligands, radioisotopes, and chelating systems currently most available for clinical PRRT. The table is functional to our objectives but does not represent the panorama of radiopharmaceuticals developed for diagnostic or therapeutic use and currently subjected to preclinical or clinical studies. (DTPA: diethylenetriamine-pentaacetic acid; DOTA: 1,4,7,10-tetraaza-cyclododecane-1,4,7,10-tetraacetic acid).

SSt-As Ligands	Currently Used for…
[Phe1]-octreotide (Oc)	diagnosis
[Tyr3]-octreotide (TOC) or SMT487	diagnosis & therapy
[Tyr3,Thr8]-octreotide o [Tyr3]-octreotate (TATE)	diagnosis &therapy
[1-Nal3]-octreotide (NOC)	diagnosis
**Radioisotopes**	
γ-emitter: Indium-111 (111In)	diagnosis & dosimetry & therapy
Technetium-99 m	diagnosis
β^+^-emitters: Yttrium-86 (86Y)	dosimetry
Gallium-68 (68Ga)	diagnosis
β^−^-emitters: Yttrium-90 (90Y)	therapy
Lutetium-177 (177Lu)	dosimetry & therapy
**Chelating Systems**	
DTPA	diagnosis
DOTA	diagnosis & therapy
HYNIC	diagnosis

**Table 2:** Physical characteristics of ^90^Y and ^177^Lu [18].

^90^Y		^177^Lu	
Max Energy β^−^	2.284 MeV (99.9%)	Max Energy β^−^	497.8 keV (100%)
T_1/2_	64.1 h	T_1/2_	6.6475 d
β^−^ human tissue range (max)	11 mm	β^−^ human tissue range (max)	1.7 mm
β^−^ human tissue range (mean)	3.9 mm	β^−^ human tissue range (mean)	0.23 mm

**Table 3:** Absorbed dose (median and range of variation) for kidney and tumor lesions [27].

Radiopharmaceuticals	Tumor Lesions (mGy/MBq)	Kidneys (mGy/MBq)
[177Lu]Lu-DOTATOC	4.9 (0.3–39.7)	0.6 (0.3–1.6)
[177Lu]Lu-DOTATATE	5.2 (0.1–89.6)	0.8 (0.3–2.6)
[177Lu]Lu-DOTANOC	2.0 (0.5–31.7)	1.1 (0.6–1.5)

**Table 4:** The main studies in which the radiopeptide [^177^Lu]Lu-DOTATATE was used for the treatment of neuroendocrine tumors (NETs), gastro-entero-pancreatic tract (GEP-NETs) and other histotypes.

Autor	Patients	Therapy Response	Survival	Toxicity	Note
Kwekkeboom DJ, 2003, [63]	n. 35 GP-NETs	CR: 3%		Nausea and vomiting within the first 24 h after administration in 30% of pts; WHO toxicity grade 3 anaemia, leucocytopenia and thrombocytopenia: 0%, 1% and 1%; Serum creatinine and creatinine clearance did not change significantly.	Patients were treated with doses of 100, 150 or 200 mCi [^177^Lu]Lu-octreotate, to a final cumulative dose of 600–800 mCi, with treatment intervals of 6–9 weeks
		PR: 35%			
		SD: 41%			
		PD: 21%			
Kwekkeboom DJ, 2008, [62]	n. 310 GEP-NETs	CR: 2%	PFS: 33 m OS: 46 m	Hematologic toxicity G3–4: 3.6% MDS: 3 pts; Temporary, nonfatal, liver toxicity: 2 Pts.	Few adverse events, interesting efficacy
		MR 15%			
		PR: 28%			
		SD: 35%			
		PD: 20%			
Sward C, 2010, [64]	n. 26 GEP-NETs	MR + PR: 37%		G3 hematologic toxicity: 3 pts; A significant reduction in GFR (*p* = 0.0013) was observed during follow-up.	Authors underline the role of absorbed dose to the kidneys as a limiting factor for PRRT.
		SD: 50%			
		PD: 13%			
Garkavij M, 2010, [65]	n. 12 NETs	OR: 17%		Transitory nausea G2: 30%; Vomiting G2: 10%; Abdominal pain G2:6%; Neutropenia G3: 2 pt; Thrombocytopenia G3: 2 pt.	Renal and hematologic toxicity did not indicate any restriction for a more aggressive approach.
		MR: 25%			
		SD: 41%			
		PD: 17%			
Bodei L, 2011, [66]	n. 51 NETs	OR: 32.6%	PFS: 36 m	Leucopenia G3: 1 pts; OS at 36 mts: 68%; Thrombocytopenia G3: 1 pts; Median creatinine clearance decrease after PRRT: 6 mts 21.7%, 1 year 23.9%, 2 years 27.6%.	Treatment was well-tolerated and effective.
		CR: 2%			
		PR: 27%			
		MR + PR: 26%			
		SD: 27%			
		PD: 18%			
Sowa-Staszczak A, 2011, [67]	n. 46 NETs	PR: 31%	PFS: 37.4 m	During 12 months follow-up, transient decrease of PLT, WBC and haemoglobin values was observed; A transient increase of creatinine level (within normal ranges) and decrease of GFR were found.	NETs [^90^Y]Y-octreotate therapy results in symptomatic relief and tumour mass reduction; The mild critical organ toxicity does not limit the PRRT of NETs.
		SD: 47%			
		PD: 22%			
van Vliet EI, 2012, [68]	n. 42 NETs	DCR: 83%			The tumor markers also decreased significantly after treatment.
van Vliet EI, 2013, [69]	n. 268 NETs	OR: 28%	PFS: 34 m OS: 74 m	Pts with PD as treatment outcome had significantly shorter PFS and OS than patients with an OR or stable disease with all 4 scoring systems; PFS and OS were comparable for pts with tumor regression and stable disease.	Both Response Evaluation Criteria In Solid Tumors (RECIST) (unidimensional) and Southwest Oncology Group (SWOG) solid tumor response criteria (bidimensional) were considered.
		SD: 49%			
		PD: 24%			
Sansovini M, 2013, [70]	n. 52 P-NETs	DCR: 81%		The most common AEs were transient hematologic toxicity, nausea, asthenia, and mild alopecia (max G2); G3 renal toxicity: 1 pts.	PFS was significantly longer after a total activity of 27.8 GBq, which can thus be considered the recommended dosage
Ezzeddin S, 2014, [71]	n. 74 GEP-NETs	MR: 17.6%	PFS: 26 m OS: 55 m	G3–4 transient myelosuppression: 10%; No irreversible toxicity, including renal toxicity (G3–4), was noted.	Even patients with a Ki-67 index ofg reater than 10% seemed to benefit from PRRT.
		PR: 36.5%			
		SD: 35.1%			
		PD: 10.8%			
Paganelli G, 2014, [72]	n. 43 GE-NETs	DCR: 84%	PFS: 36 m	The most common AEs were transient hematologic toxicity, nausea, asthenia, and mild alopecia (max G2).	Both activity levels (27.8 GBq and 18.5 GBq) proved safe and effective in all pts.
		CR: 7%			
		SD: 77%			
Delpassand ES, 2014, [73]	n. 37 GEP-NETs	MR: 3%		No significant acute or delayed hematologic or renal toxicity was observed.	Treatment was well-tolerated and effective, QOF was also improved.
		PR: 28%			
		SD: 41%			
		PD: 28%			
Danthala M, 2014, [74]	n. 40 NETs	MR: 25%		Mild renal toxicity: 1 pt; Carcinoid crisis: 1 pt; Fatal hepatic failure: 1 pt; Myocardial infarction 2 mts after the second cycle: 1 pt.	Best responses were reported when more than 2 cycles were given; Accurate safety controls requested.
		PR: 32.5%			
		SD: 22.5%			
		PD: 20%			
Sabet A, 2015, [75]	n. 61 GE-NETs	DCR: 91.8%	PFS: 33 m OS: 61 m	Reversible hematologic toxicity (≥G3): 8.2%; No significant renal toxicity (≥G3) was Observed.	High DCR and long PFS can be achieved with PRRT after failure of standard biotherapy.
		MR: 31.1%			
		PR: 13.1%			
		SD: 47.5%			
		PD: 8.2%			
van Vliet EI, 2015, [76]	n. 29 P-NETs		PFS: 69 m	The median PFS was 69 mo for patients with successful surgery and 49 mo for the other patients; For comparison, the median PFS in 90 other patients with a nonfunctioning pancreatic NET with more than 3 liver metastases or other metastases was 25 mo.	After the treatment with [^177^Lu]Lu-octreotate, successful surgery was performed in 9 of 29 patients (31%); Neoadjuvant treatment with [^177^Lu]Lu-octreotate is a valuable option for patients with initially unresectable pancreatic NETs.
Strosberg J, 2017, [25]	n. 116 + 113 GE-NETs		OR: 18%	At the cut off date for the primary analysis, the estimated rate of progression-free survival at month 20 was 65.2% in the [^177^Lu]Lu-octreotate group and 10.8% in the control group.	First phase 3 study: PRRTversus LAR octreotide

Legend: DCR: disease control rate, OR: overall response, CR: complete response, MR: ninor response, PR: partial response, SD: stable disease, PD: progressive disease, PFS: progression-free-survival, OS: overall serviva, NETs: neuroendocrine tumours, GEP: gastroenteropancreatic, P: pancreatic, GE: gastroenteric.

**Table 5:** The main studies in which the radiopeptide [^177^Lu]Lu-DOTATOC was used for the treatment of NETs and other histotypes.

Autor	Patients	Therapy Response	Survival	Toxicity	Note
Forrer F, 2005, [77]	n. 27 NETs	MR: 18%		Therapy was well-tolerated. No serious adverse events occurred	[^177^Lu]Lu-DOTATOC therapy in patients with relapse after [^90^Y]Y -DOTATOC treatment is feasible, safe, and efficacious
		PR: 7%			
		SD: 44%			
		PD: 30%			
Baum RP, 2016, [26]	n. 56 NETs	DCR: 66.1%	PFS: 17.4 OS: 34.2	There were no serious AEs; One case of self-limiting G3 myelotoxicity was reported; Although 20% of pts had mild renal insufficiency at baseline, there was no evidence of exacerbated or de novo renal toxicity after PRRT.	The observed safety profile suggests a particularly favorable therapeutic index, including in patients with impaired bone marrow or renal function.
		OR: 33.9%			
		CR: 16.1%			
Frilling A, 2006, [78]	n. 20 NETs	PR: 25%		After ^90^Y treatment moderate toxicity was observed in 8 patients; No serious adverse events were documentable.	[^177^Lu]Lu-DOTATOC and [^90^Y]Y-DOTATOC treatment; All patients received [^90^Y]Y-DOTATOC as initial treatment. In case of disease relapse the treatment was repeated with [^177^Lu]Lu-DOTATOC.
		SD: 55%			
		PD: 20%			
Forrer F, 2008, [79]	n. 28 PGG/FCC	PR: 7%	PFS: 3–42 m	Authors found 1 thrombocytopenia grade 1, and 1 anemia grade 1; No non-hematological toxicity, especially no kidney toxicity occurred.	[^177^Lu]Lu-DOTATOC and [^90^Y]Y-DOTATOC treatment; Therapy seems to be less effective than in gastroentero-pancreatic neuroendocrine tumors.
		MR: 25%			
		SD: 47%			
		PD: 21%			
Pfeifer AK, 2011, [80]	n. 69 NETs	CR: 7.4%	PFS: 29 m	The overall frequency of serious adverse events was low.	[^177^Lu]Lu-DOTATOC or [^90^Y]Y-DOTATOC treatment; Pancreatic NET seemed to respond better to PRRT than small intestinal carcinoid tumors (*p* = 0.03).
		PR: 16.2%			
		SD: 61.8%			
		PD: 14.4%			
Villard L, 2012, [81]	n. 486 NETs		OS: 3.96–5.51 y	The rates of severe hematologic toxicities (6.3% v 4.4%; *p* = 0.25) and severe renal toxicity (8.9% v 11.2%; *p* = 0.47) were comparable in both groups.	[^177^Lu]Lu-DOTATOC or/and [^90^Y]Y-DOTATOC treatment; Patients receiving [^90^Y]Y-DOTATOC + [^177^Lu]Lu-DOTATOC had a significantly longer survival than patients receiving [^90^Y]Y-DOTATOC alone (5.51 v 3.96 years; hazard ratio, 0.64; 95% CI, 0.47 to 0.88; *p* = 0.006).
Dumont RA, 2014, [82]	n. 36 GSN	OR: 72.2%	OS: 45.1 m	A total of 21 patients (58.3%) experienced hematotoxicity grade 1/2, while 1 patient (2.8%) experienced hematotoxicity grade 3; no grade 4 hematotoxicity occurred; Furthermore, 2 patients (5.6%) developed grade 4 renal toxicity; no grade 5 renal toxicity occurred.	[^177^Lu]Lu-DOTATOC and [^90^Y]Y-DOTATOC treatment; Response to [^90^Y]Y-DOTATOC and [^90^Y]Y-DOTATOC plus ^177^Lu]Lu-DOTATOC therapy is associated with a longer survival in patients with metastasized gastrinoma.
Romer A, 2014, [83]	n. 1051 NETs		OS: 35.9–45.5 m	The rate of severe transient haematotoxicities was lower after [^177^Lu]Lu-DOTATOC treatment (1.4 vs. 10.1%, *p* = 0.001), while the rate of severe permanent renal toxicities was similar in both treatment groups (9.2 vs. 7.8%, *p* = 0.32)	[^177^Lu]Lu-DOTATOC or [^90^Y]Y-DOTATOC treatment; The present results revealed nodifference in median overall survival after [^177^Lu]Lu-DOTATOC and [^90^Y]Y-DOTATOC;Furthermore, [^177^Lu]Lu-DOTATOC was less haematotoxic than [^90^Y]Y-DOTATOC.
Marincek N, 2015, [84]	n. 34 MNG	SD: 68%	OS: 8.6 y	Severe hematotoxicity occurred in 3 patients, and severe renal toxicity in 1 patient.	[^177^Lu]Lu-DOTATOC and [^90^Y]Y-DOTATOC treatment; [^177^Lu]Lu-DOTATOC and [^90^Y]Y-DOTATOC are promising tools for treating progressive unresectable meningioma, especially in cases of high tracer uptake in the tumor.
Rodojewski P, 2015, [85]	n. 1449 NETs	OR: 39.3–53.8%	OS: 48.2–64.9 m	[^90^Y]Y-DOTATOC induced higher hematotoxicity rates than combined treatment (9.5% vs. 4.0%, *p* = 0.005) or [^177^Lu]Lu-DOTATOC (9.5 vs. 1.4%, *p* = 0.002). Renal toxicity was similar among the treatments.	[^177^Lu]Lu-DOTATOC or/and [^90^Y]Y-DOTATOC treatment; [^90^Y]Y-DOTATOC plus [^177^Lu]Lu-DOTATOC was associated with longer survival than [^90^Y]Y-DOTATOC or [^177^Lu]Lu-DOTATOC alone
Horsch D, 2016, [86]	n. 450 NETs	DCR: 66.1%	PFS: 41 m OS: 38 m	Bone marrow and renal function AEs higher than G3: 0.2–1.5%.	[^177^Lu]Lu-DOTATOC or/and [^90^Y]Y-DOTATOC treatment; Treatment was well-tolerated and effective

Legend: DCR: disease control rate, OR: overall response, CR: complete response, MR: ninor response, PR: partial response, SD: stable disease, PD: progressive disease, PFS: progression-free-survival, OS: overall serviva, NETs: neuroendocrine tumours, PGG: Paragangliomas, FCC: Pheocromicytomas, GSN: Gastrinomas, MNG: Meningiomas.

## Data Availability

Not applicable.
